# Poly(methyl vinyl ether-*alt*-maleic acid) and ethyl monoester as building polymers for drug-loadable electrospun nanofibers

**DOI:** 10.1038/s41598-017-17542-4

**Published:** 2017-12-08

**Authors:** Amalia Mira, C. Reyes Mateo, Ricardo Mallavia, Alberto Falco

**Affiliations:** Universidad Miguel Hernández (UMH), Instituto de Biología Molecular y Celular (IBMC), 03202 Elche (Alicante), Spain

## Abstract

New biomaterials are sought for the development of bioengineered nanostructures. In the present study, electrospun nanofibers have been synthesized by using poly(methyl vinyl ether-alt-maleic acid) and poly(methyl vinyl ether-alt-maleic ethyl monoester) (PMVEMA-Ac and PMVEMA-ES, respectively) as building polymers for the first time. To further functionalize these materials, nanofibers of PMVEMA-Ac and PMVEMA-ES containing a conjugated polyelectrolyte (HTMA-PFP, blue emitter, and HTMA-PFNT, red emitter) were achieved with both forms maintaining a high solid state fluorescence yield without altered morphology. Also, 5-aminolevulinic acid (5-ALA) was incorporated within these nanofibers, where it remained chemically stable. In all cases, nanofiber diameters were less than 150 nm as determined by scanning and transmission electron microscopy, and encapsulation efficiency of 5-ALA was 97 ± 1% as measured by high-performance liquid chromatography. Both polymeric matrices showed rapid release kinetics in vertical cells (Franz cells) and followed Higuchi kinetics. In addition, no toxicity of nanofibers, in the absence of light, was found in HaCaT and SW480 cell lines. Finally, it was shown that loaded 5-ALA was functional, as it was internalized by cells in nanofiber-treated cultures and served as a substrate for the generation of protoporphyrin IX, suggesting these pharmaceutical vehicles are suitable for photodynamic therapy applications.

## Introduction

Nanotechnology has gained new impetus recently thanks to the solutions it offers for both the development of new biomedical applications and the improvement of conventional ones^1–3^. To a large degree, these advances are due to the development of novel materials that allow the design of nanostructures with physicochemical properties that could overcome the limitations that hinder further progress in biomedicine^4–7^. The multiple design possibilities in both (nano)size and (nano)structure presented by this technology make it a very versatile tool with potential for use in various applications and with the ability to be incorporated into almost any biomedical device^6,8^. In addition, this technology offers new advantageous uses because of the properties that emerge only when molecules are nanoscopically arranged; properties not present in their macroscopic arrangement^9^.

Among the many applications that nanotechnology can contribute to, nanoencapsulation can add significantly to the protection of pharmaceutical agents against environmental degradation, to site-specific targeted delivery and/or to controlled release^1,10^. From a clinical point of view, these contributions are of great interest because they might improve the efficiency of the therapeutic and diagnostic compounds by, for instance, increasing their circulating half-lives and specificity, and thus reducing the necessary dosage and side-effects^3,5,6^.

As to the materials that can be used for this purpose, polymers have the advantage of comprising a large variety of biocompatible and biodegradable molecules, including many suitable for the construction of different types of drug-loadable nanostructures that meet clinical needs, through relatively facile and short protocols^4,11–14^. By using appropriate polymer/s, such assemblies can be designed, for instance, to contain molecules of very different nature, size and water solubility and to control their release and maintain concentrations at therapeutically relevant levels for prolonged periods^8,15^. Besides, they can be shaped and size-adjusted to match the requirements of the delivery route/device of choice. With regard to this latter feature, the most popular forms, while versatile, are mainly nanoparticulated ones, although polymer nanofibers are becoming increasingly important because of their unique suitability to medical applications such as tissue scaffolds and dressings^4,15–17^.

The size scale of the nanofibers, similar to that of cellular components, can mimic that of the extracellular matrix of biological tissues, and interactions at the molecular level could be exploited to enhance the therapeutic potential of functional agents loaded within nanofibers^4,16,17^. Different techniques including vapor deposition^18,19^, phase separation^11,20,21^, self-assembly^22–24^ and centrifugal spinning^25–27^ can be used to synthesize such nanofibers, but electrospinning is the most widely adopted one due to its advantages for implementation/commercialization purposes^28–31^.

In general, and in contrast to other techniques, the fabrication of nanofibers by electrospinning is simple, cost-effective and scalable^30,31^. Electrospun polymer nanofibers are also highly tunable and it is possible to produce 3D structures through simple mechanical modifications in the set-up^17^. In addition, for drug release applications in particular^12^, nanofibers generated by electrospinning are highly amenable to loading with molecular cargo, up to 60% w/w in some cases^30^, and encapsulation yields close to full efficiency^32^.

Another factor that contributes to the versatility and flexibility of the technique is the possibility of using both natural and synthetic electrospinnable polymeric compounds, individually or by combining different polymers, in order to achieve nanofibers with desired physico-chemical and, consequently, functional properties^4^. Therefore, selecting the most suitable polymeric materials is critical and characterizing a wide variety of compounds is of great benefit to the bionanotechnology community.

Copolymers of alternating methyl vinyl ether and maleic anhydride (poly-methyl vinyl ether-alt-maleic anhydride, PMVE/MA) and its derived forms, which are sold commercially under the generic trademark Gantrez, are biodegradable molecules that have shown low toxicity and high biocompatibility and display useful bioadhesive properties^33–38^. These synthetic compounds have been used for medical purposes including encapsulation of pharmaceutical agents in nanoparticles^33–36^. Further, electrospun nanofibers made of blends of polymers including PMVE/MA with loading capacity have recently been reported^39,40^.

Here, two forms of PMVE/MA (PMVE/MA-Ac and PMVE/MA-ES) polymeric material were assessed for the capacity to create drug-loadable electrospun nanofibers. Also, the distribution of a potential load along the electrospun film was analyzed by loading with either a red- or blue-emitting fluorene-based cationic polyelectrolyte (CPE), as a fluorescent marker. Further, the encapsulation capacity of these electrospun nanofibers was assessed using a clinically-relevant pharmaceutical agent, the photosensitizer 5-aminolevulinic acid (5-ALA), which is widely used in photodynamic therapy (PDT). Its release dynamics and compound stability are analyzed for all combinations of polymeric nanofibers mentioned above (PMVE/MA forms ± CPEs).

## Results

### Morphology and size of nanofibers of PMVE/MA forms

Initial efforts were focused on the optimization of the conditions to obtain electrospun nanofibers of PMVE/MA derivatives. The concentration of each polymer form seemed to be critical for achieving nanofibers with uniform size and shape. Briefly, preliminary optimization studies performed in our lab led to the selection of the general electrospinning settings described in the materials and methods section, including a polymer concentration of 20% w/w for the initial electrospinning solutions. As a result, nanofibers were obtained with both PMVE/MA forms, but only those based on PMVE/MA-Ac met proposed quality standards (ie continuous nanofibers with uniform size and shape and no bifurcations or thickenings), whereas PMVE/MA-ES ones were irregular and with beads (Supplementary Fig. S1A). Since this eventuality is often due to low viscosity levels, higher PMVE/MA-ES concentrations (with corresponding higher viscosity rates) were tried. Among them, nanofibers with desired features were obtained by using 25% w/w PMVE/MA-ES solutions (Supplementary Fig. S1B), while at concentrations of 30% w/w they were again not suitable because of larger nanofiber diameter (more than 1 µm diameters) and systematic fusion events (Supplementary Fig. S1C).

**Figure 1:**
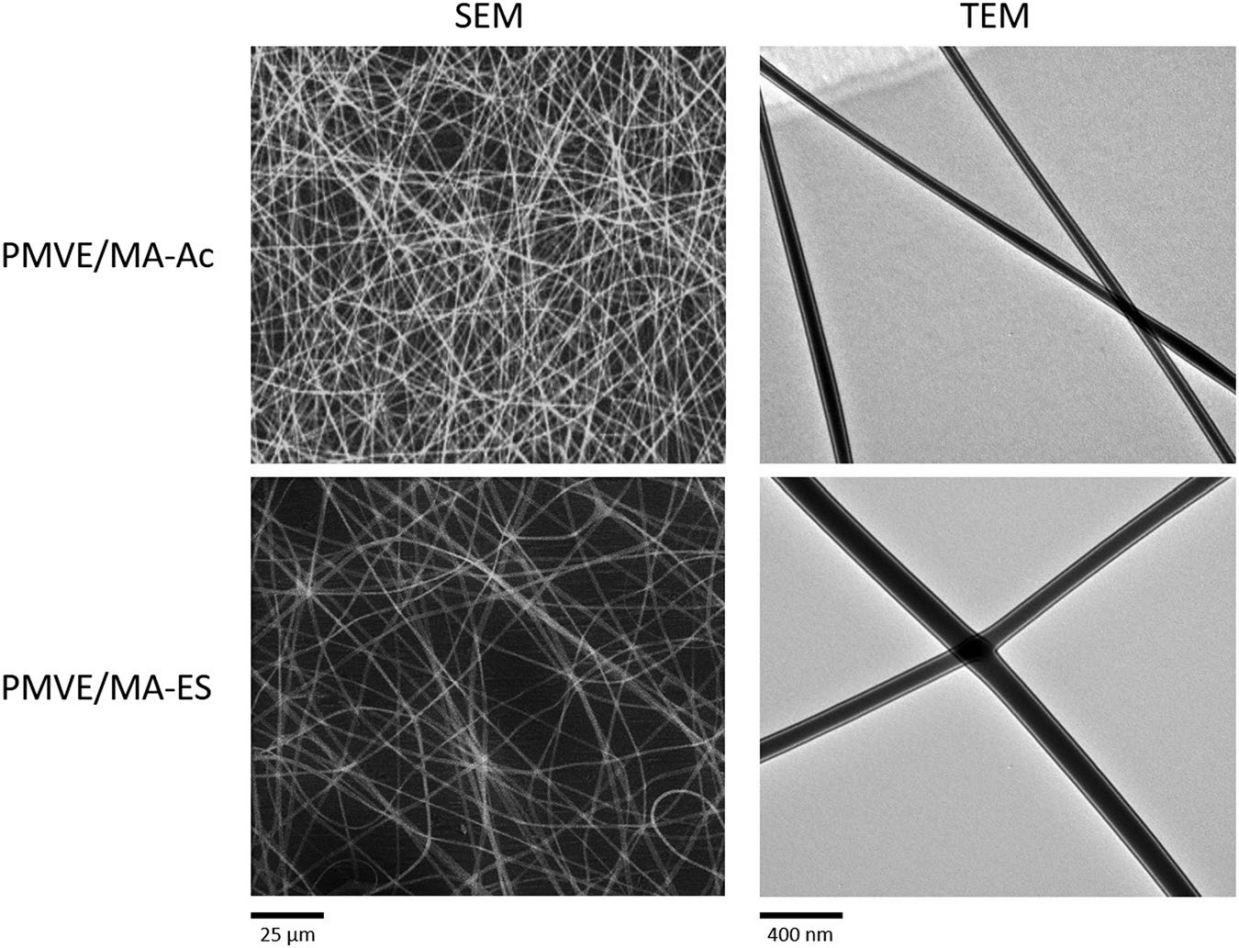
SEM and TEM images of different polymer nanofiber systems. Representative images of the polymer nanofibers obtained from 20% w/w PMVE/MA-Ac solutions in water and 25% w/w PMVE/MA-Ac solutions in ethanol.

Representative scanning and transmission electron micrographs (SEM and TEM, respectively) of nanofibers synthesized under optimal conditions with each PMVE/MA form are shown in Fig. 1. All the fabricated mats showed randomly oriented nanofibers with a uniform surface appearance. The diameters of these nanofibers, calculated from TEM images of different mat batches (n = 3), varied slightly depending on the polymeric source used, being 55 ± 5 nm for PMVE/MA-Ac and 60 ± 4 nm for PMVE/MA-ES nanofibers.

### Morphology of nanofibers of single and CPE-blended PMVE/MA forms solutions w/ or w/o 5-ALA

Subsequently, the morphology of nanofibers synthesized under the above conditions but using a polymer blend with the PMVE/MA derivatives as base compounds was described. The chosen companion polymers were fluorene-based CPEs to function as reporters of successful inclusion, detectable through their fluorescent properties. This feature helps to assess the ability of a base polymer to accumulate properties of other compounds in the final nanostructure as well as to check the homogeneity of the blend throughout the entire synthesis process.

**Figure 2:**
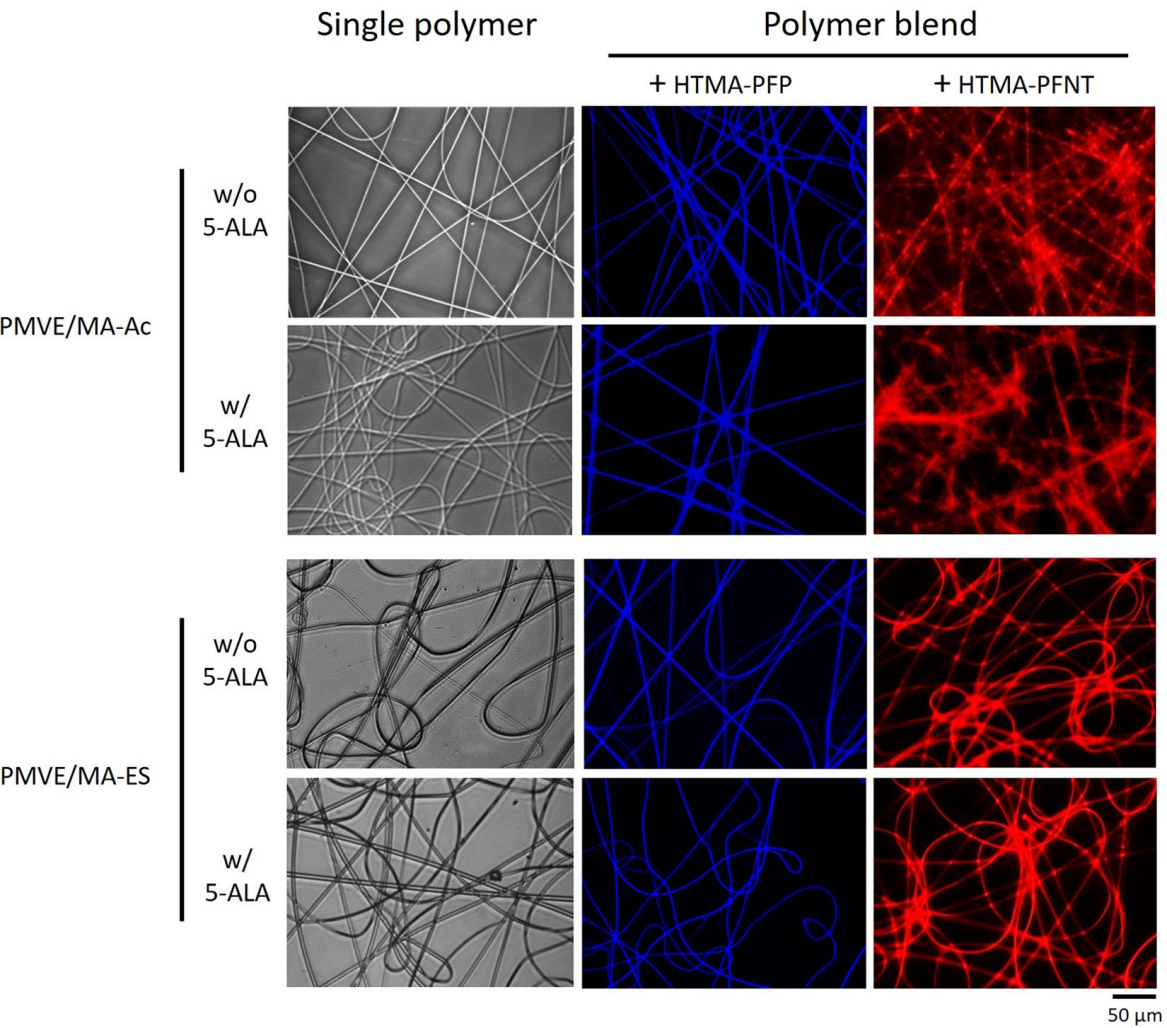
Fluorescence microscopy of different polymer nanofiber systems. Images include detail of electrospun nanofibers from either 20% w/w PMVE/MA-Ac solutions in water or 25% w/w PMVE/MA-ES solutions in ethanol (single polymer), blends of these solutions with CPEs HTMA-PFP (blue nanofibers) or HTMA-PFNT (red nanofibers) and all mentioned combinations w/ or w/o 5-ALA (16.7% w/w).

Optical micrographs of electrospun nanofibers made with all polymer combinations, w/ or w/o 16.7% w/w 5-ALA, are shown in Fig. 2. Loaded 5-ALA was detected by high performance liquid chromatographic (HPLC) and its average encapsulation yield calculated, reaching 97 ± 1% (n = 4). Morphological differences were only apparent when comparing the polymer source used. Blends of any PMVE/MA derivate with HTMA-PFP allowed the synthesis of uniform nanofibers with a highly homogeneous distribution of the fluorescence. Similar characteristics were observed in nanofibers obtained from blends of PMVE/MA-ES and HTMA-PFNT, but this contrasted with the non-uniform structural pattern and fluorescence distribution shown by those nanofibers made of a blended polymer solution including PMVE/MA-Ac and HTMA-PFNT.

### Stability of CPEs in nanofiber solutions blended from PMVE/MA derivatives

**Figure 3:**
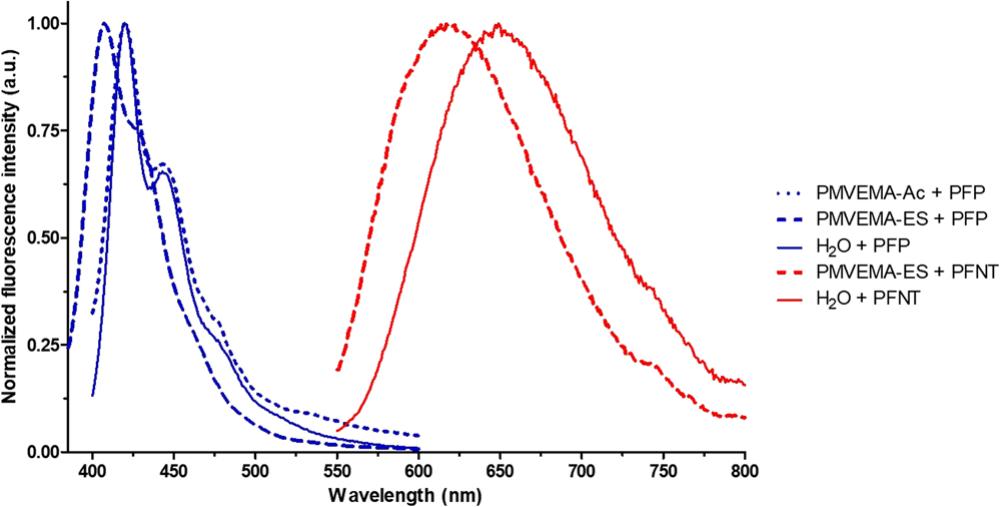
Fluorescence spectroscopy. Normalized fluorescence spectra of HTMA-PFP (blue lines, +PFP) and HTMA-PFNT (red lines, +PFNT) in water solution (solid lines) and electrospun blended solutions with 20% w/w PMVE/MA-Ac (dotted lines) and from 25% w/w PMVE/MA-ES (dashed lines).

Changes in the fluorescence spectra of a compound are indicators of its stability. Thus, they were recorded for all polymer blend solutions that generated homogeneous nanofibers. Fluorescence spectra for these blends (Fig. 3) were similar to the corresponding spectra for CPE in water, but displaced to lower wavelengths. The only exception was the spectrum of the solution combining HTMA-PFP with PMVE/MA-Ac, which matched its control quite closely. Thus, HTMA-PFP either in water, or combined with PMVE/MA-Ac, exhibited a main peak at 421 nm and a secondary one at 443 nm, whereas HTMA-PFP-blended PMVE/MA-ES solution peaked at 407 nm and showed a smooth shoulder-like shape at about 430 nm. Regarding the fluorescence spectrum of HTMA-PFNT-blended PMVE/MA-ES solution, it showed a unique peak at 601 nm, 29 nm lower than the also unique one corresponding to HTMA-PFNT in water.

### Transdermal release dynamics of 5-ALA from single and CPE-blended PMVE/MA nanofibers

**Figure 4:**
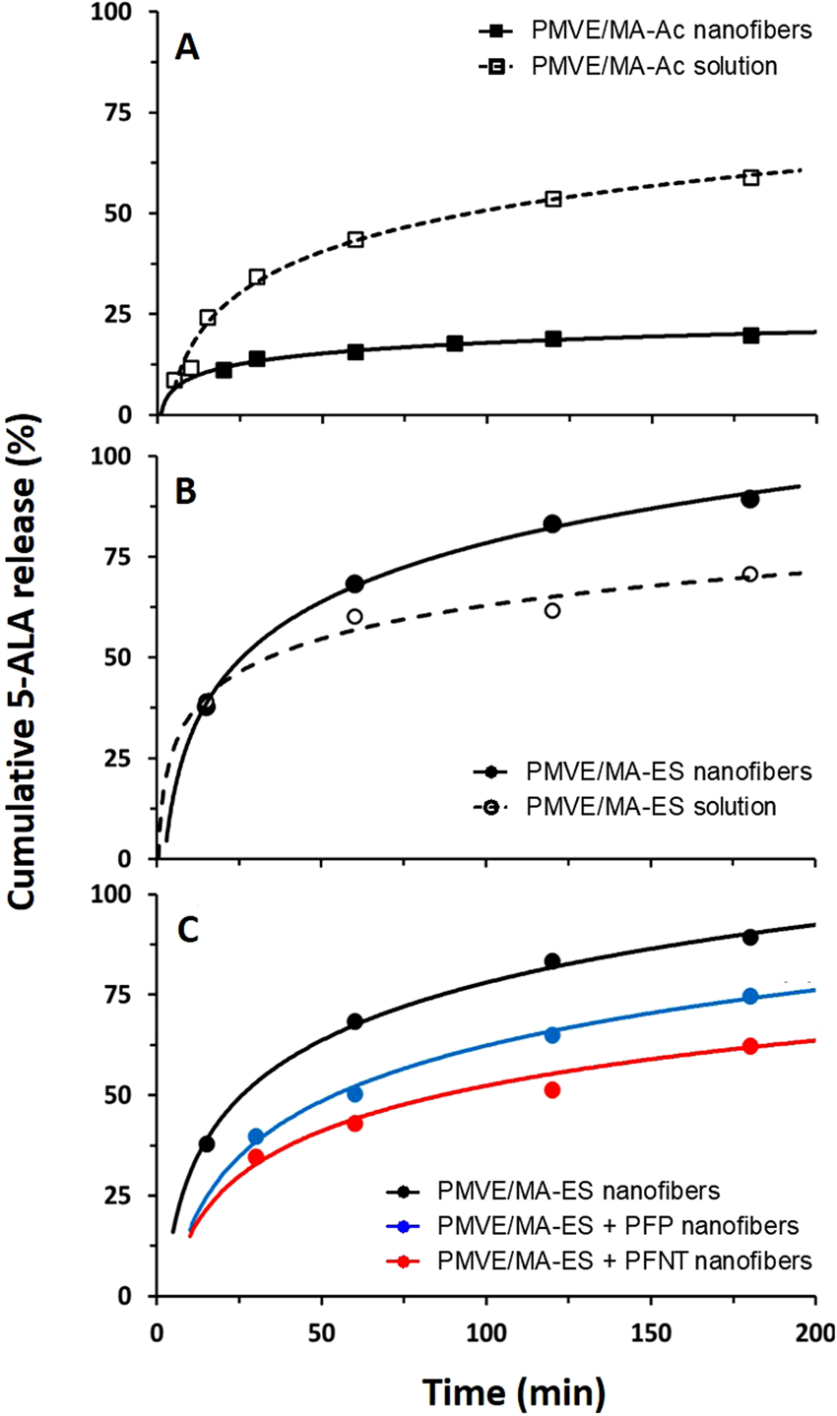
Release profile of 5-ALA loaded into nanofibers. (**A**) 5-ALA loaded into PMVE/MA-Ac nanofibers (full squares, solid line) and its solution before electrospinning (empty squares, dashed line). (**B**) 5-ALA loaded into PMVE/MA-ES nanofibers (full circles, solid line) and its solution before electrospinning (empty circles dashed line). (**C**) 5-ALA loaded into electrospun nanofibers made of PMVE/MA-ES (black line), a PMVE/MA-ES-HTMA-PFP (+PFP) blend (blue line) and a PMVE/MA-ES-HTMA-PFNT (+PFNT) blend (red line). In all cases: n = 2, sd < 10%.

To evaluate the potential of electrospun nanofibers for topical release of transdermal therapeutics, the release dynamics of encapsulated 5-ALA were studied in Franz cells. Initially, this *in vitro* transdermal release methodology was used to analyze single 5-ALA-loaded nanofibers composed of PMVE/MA-Ac (Fig. 4A) and PMVE/MA-ES (Fig. 4B). Results were compared to corresponding control samples, ie raw polymer solutions (not electrospun) including 16.7% w/w 5-ALA.

HPLC analysis of samples taken from the collector at multiple time-points revealed that 5-ALA transdermal delivery kinetics were different depending on the PMVE/MA variant used (Fig. 4A,B). For PMVE/MA-Ac (Fig. 4A), nanofibers released barely 20% of total encapsulated 5-ALA at the latest time-point checked (3 h), with the release curve showing a plateau starting at about 2 h. In contrast, non-electrospun PMVE/MA-Ac solution produced 60% drug release at 3 h, having released 50% of total drug content at about 95 min. On the other hand, PMVE/MA-ES not only achieved higher rates of transdermal drug release independently of the form tested (either electrospun or in solution), but also this release seemed to be more efficient when nanostructured as an electrospun nanofiber (90% of content release in 2 h) than in solution (70% of content release in 2 h) (Fig. 4B).

In order to assess whether polymer blends might improve upon the highly efficient release rates shown by PMVE/MA-ES nanofibers, 5-ALA release dynamics were also studied for CPE-blended PMVE/MA-ES nanofibers (Fig. 4C). CPEs modified release curves by reducing the amount of 5-ALA liberated without altering the general dynamic profile. Thus, HTMA-PFP- and HTMA-PFNT-blended nanofibers showed, respectively, 70 and 60% cumulative 5-ALA release at 3 h.

Attempts to match obtained data to four classical kinetic release models are shown in Table 1 and it was found that when nanostructured as fibers the content diffuses preferentially according to Fick’s law (Higuchi model in particular).

**Table 1:** Comparative of the kinetic values among different release models for the experimental formulations of the study.

Polymer		Zero order		First order		Higuchi		Korsmeyer-Peppas		
Type	Form	K_0_	R^2^	K_1_	R^2^	K_H_	R^2^	K	n	R^2^
PMVE/MA-Ac	Dissolved	3.0	0.85	0.19	0.54	7.16	0.93	**15.7**	**0.25**	**0.95**
	NF	14.2	0.79	0.45	0.80	**32.6**	**0.92**	40.8	0.52	0.92
PMVE/MA-ES	Dissolved	12.2	0.73	0.25	0.72	25.2	0.84	**55.5**	**0.23**	**0.92**
	NF	17.9	0.86	0.22	0.75	**42.1**	**0.95**	64.0	0.35	0.98
	NF + PFP	16.0	0.93	0.32	0.84	**41.3**	**0.98**	46.3	0.51	0.96
	NF + PFNT	9.5	0.98	0.19	0.94	**26.4**	**0.99**	43.0	0.32	0.99

Best adjustments (highest R^2^ with less than 60% conversions) are indicated in bold.

NF, nanofibers; PFP, HTMA-PFP; PFNT, HTMA-PFNT.

### Determination of the cell viability after treatment with electrospun PMVE/MA-ES nanofibers

**Figure 5:**
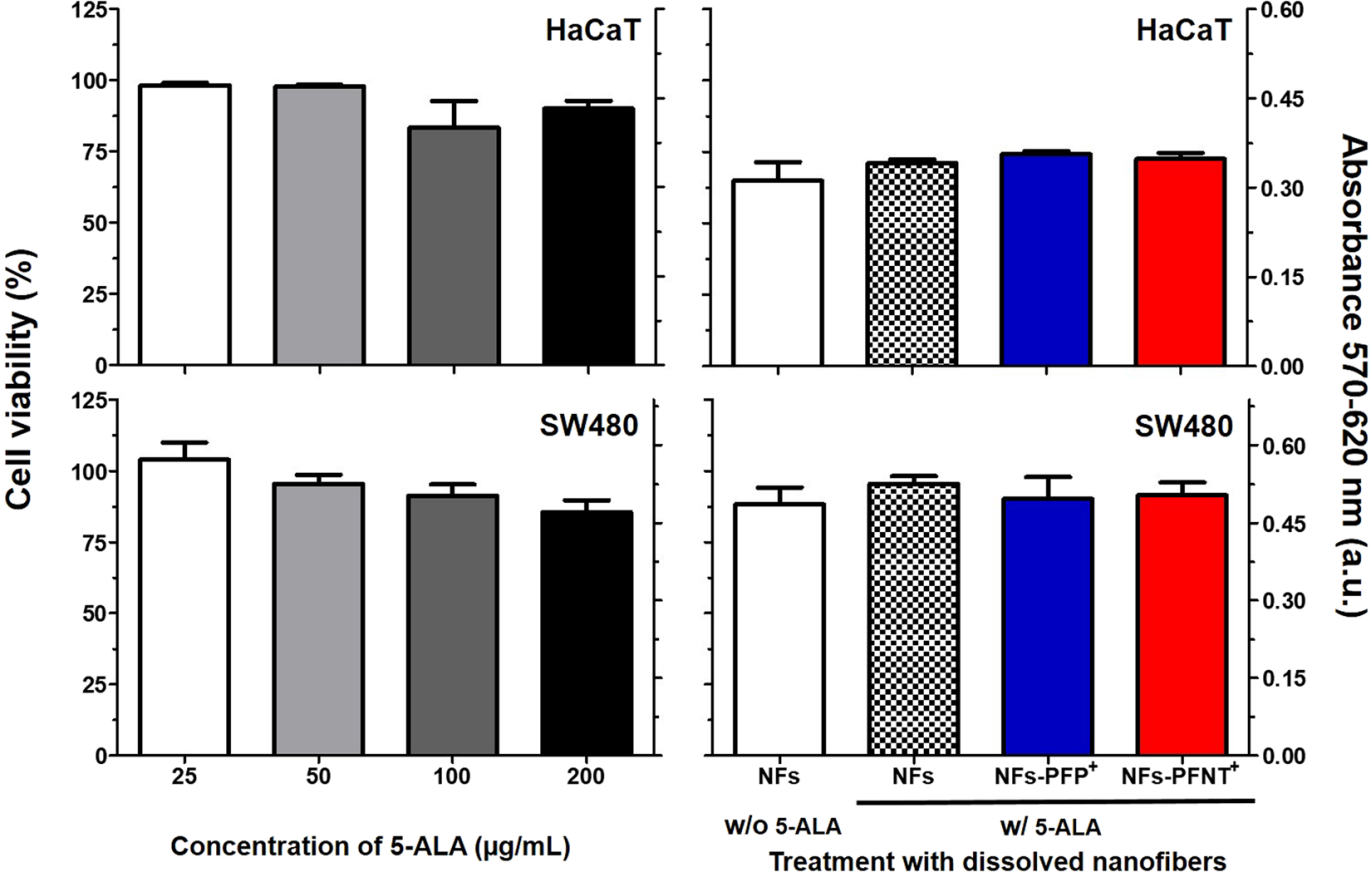
Viability of HaCaT and SW480 cells treated with nanofiber-loaded and non-loaded 5-ALA. Treatments include a gradient of 5-ALA concentrations and a solution of different types of PMVE/MA-ES electrospun nanofibers (6 mg/mL of PMVEMA-ES). Cell viability is shown in % relative to non-treated control cells (left Y axis) and a.u. from optical density at 570–620 nm (right X axis) as the average (±sd) from three independent experiments performed by triplicate.

The possible toxicity induced in cells treated with electrospun PMVE/MA-ES nanofibers was also evaluated. For these assays, HaCaT and SW480 cells were treated with a single concentration of each type of 5-ALA-loaded PMVE/MA-ES nanofibers previously dissolved in PBS (6 mg/mL in PMVE/MA-ES, corresponding to 100 μg/mL of encapsulated 5-ALA). Likewise, treatment with non-loaded (no 5-ALA) PMVE/MA-ES nanofibers was also included. Different concentrations of 5-ALA were also tested to perform a calibration curve for this compound. After 24 h, cell viability was determined by Thiazolyl Blue Tetrazolium Bromide (MTT) assay as described in the material and methods section (Fig. 5).

Increasing concentration of 5-ALA was correlated with a decrease in cell viability in both cell lines. SW480 cells were slightly more sensitive to this compound than HaCaT cells (85.6 ± 4.3% versus 90.0 ± 2.8% cell viability respectively, at 200 μg/mL of 5-ALA). In none of the cases did the viability levels drop below 85% with 5-ALA treatment.

The viability values obtained for nanofiber solutions revealed that PMVEMA-ES nanofibers do not show significant toxicity levels in the SW480 cell line. For these cells, the lowest percentage of cell viability was 88.3 ± 5.9% when treated with the solution of non-loaded single polymer nanofibers. Whereas SW480 was more sensitive than HaCaT to 5-ALA, the HaCaT cell line was more sensitive to treatment with nanofibers than SW480. In this case, cell viability levels in HaCaT ranged between 64.9 ± 6.5 and 74.2 ± 1.0%, with no significant difference between treatments.

### Protoporphyrin IX (PpIX) accumulation in cells when treated with 5-ALA-loaded electrospun PMVE/MA-ES nanofibers

It might happen that the conditions during the process of synthesis of the nanofibers may degrade the compounds intended to be encapsulated. Even though no changes were found in the elution peaks of encapsulated 5-ALA in comparison to control 5-ALA samples (data no shown), thus indicating no major changes in the molecular structure, assays to ensure its activity are required. In this sense, the intracellular accumulation of PpIX in cells treated with 5-ALA from electrospun nanofibers would demonstrate the stability of the structure of the encapsulated molecule. Therefore, in an attempt to verify this issue, HaCaT and SW480 cells were treated for 24 h with a solution of 5-ALA-loaded and non-loaded PMVE/MA nanofibers in the same manner as described for determining cell viability in the previous section. CPE-blended nanofibers were not used, as their absorption and/or emission spectra overlap with PpIX (see Supplementary Fig. S2). In parallel, cells were incubated with a 5-ALA gradient in order to correlate PpIX accumulation with 5-ALA treatment concentrations. Afterwards, accumulated PpIX levels were detected as described in material and methods.

**Figure 6:**
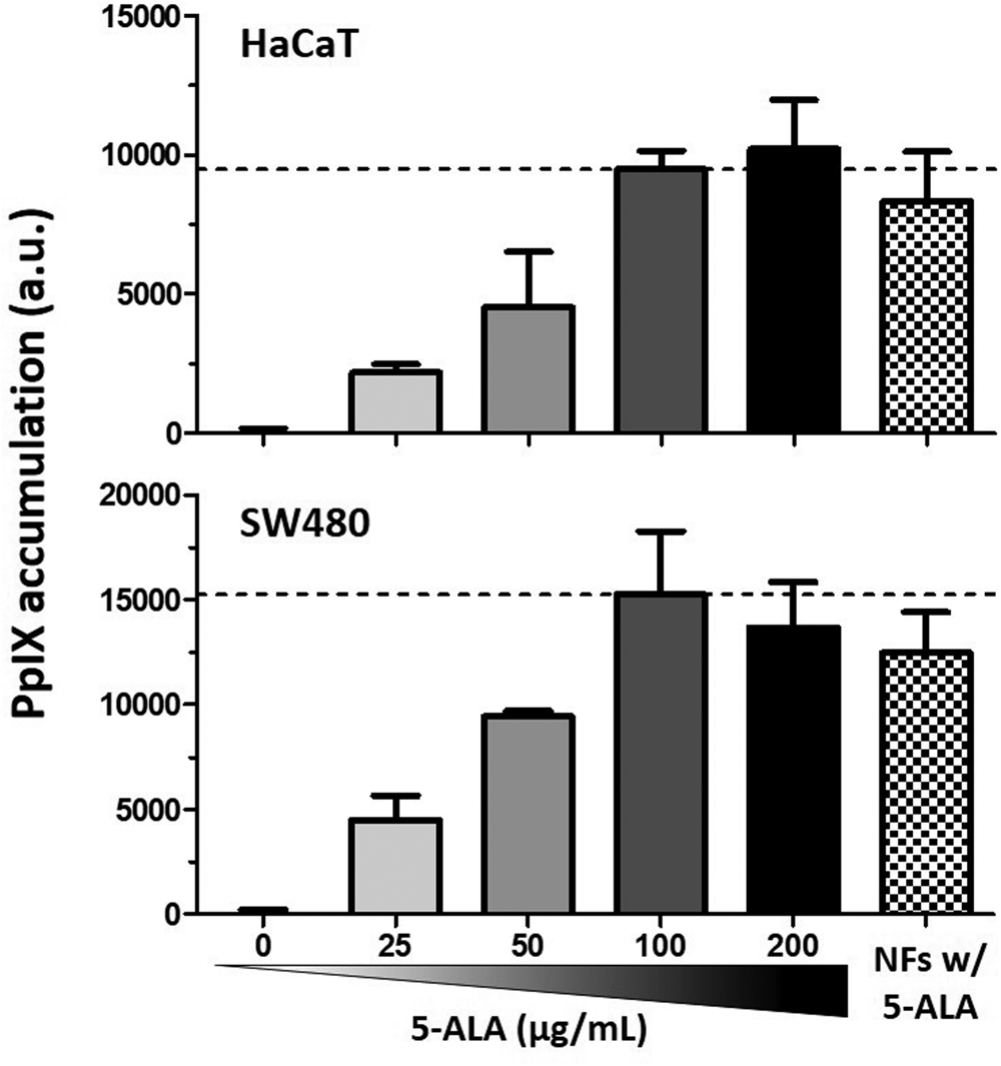
Determination of intracellular production and accumulation of PpIX after treatment with dissolved PMVE/MA-ES electrospun nanofibers. Cells were treated for 24 h with a gradient of 5-ALA concentrations and a dissolution of empty or 5-ALA-loaded PMVE/MA-ES nanofibers (6 mg/mL in PMVEMA-ES, thus 1 mg/mL of 5-ALA). No PpIX was detected for empty nanofiber treatments, so not included in graph. Samples were excited at 412 nm and emission values taken at 665 nm. Results are expressed in average a.u. (±sd) obtained from three different experiments performed by triplicate.

Results showed that this technique is very sensitive (capable of detecting even 0.013 pg of PpIX) and allows the determination of the relative amount of PpIX that is produced and accumulated in cells. Thus, increased concentrations of 5-ALA up to 100 μg/mL correlated with detected levels of PpIX, with intracellular PpIX accumulation plateauing at this concentration in both cell lines (Fig. 6). As for the detected levels of PpIX in cells treated with 5-ALA from nanofibers, these results were not significantly different (*t* test, P > 0.05) from expected ones (PpIX detection in cells treated with 100 μg/mL of non-encapsulated 5-ALA) in either of the cell lines used (Fig. 6). Specifically, by extrapolating from the 5-ALA calibration curve for each cell line, these values corresponded to 5-ALA concentrations of 88.7 ± 18.9 and 77.7 ± 12.1 μg/ml for HaCaT and SW480, respectively. Similarly, by extrapolating from the PpIX calibration curve, these values would correspond to 0.04 ± 0.009 and 0.15 ± 0.024 pg of PpIX, respectively.

### Effect of the encapsulation period on the activity of loaded 5-ALA

The present study also analyzed how the storage conditions inside the PMVE/MA-ES electrospun nanofibers affect the activity of the loaded compound over time. For this purpose, the activity of 5-ALA loaded in nanofibers from two different batches was examined in both cell lines for three months, from the moment of their production. Each time-point therefore corresponded to the mean (±sd) of two experiments performed in triplicate in both cell lines. It was observed that over the course of three months the activity of encapsulated 5-ALA remained stable and did not change significantly from when each batch was synthesized (data not shown; one-way ANOVA, P = 0.6).

## Discussion

This work is the first report of the synthesis and characterization of drug-loadable electrospun nanofibers using a PMVE/MA derivative (either PMVE/MA-Ac or PMVE/MA-ES) as the single polymer source. So far, there are only a few reports of PMVE/MA being used^39,40^, and in none of them was it employed as a single polymer source, but rather as complementary material to confer other characteristics such as biocompatibility, biodegradability or rigidity to the general nanostructure. For instance, electrospun nanofibers were made of a blend of gelatin and PMVE/MA at a 4:1 ratio with a final concentration of 10% and loaded with zinc oxide-containing nanoparticles, as part of a gelatin scaffold designed for wound healing^39^. In another study, nanofibers loaded with silver nanoparticles were produced by electrospinning a 1:1 w/w polystyrene-PMVE/MA blend and used to coat experimental membranes, providing antimicrobial properties^40^.

In our study, after optimizing general electrospinning settings, in order to synthesize nanofibers with desired morphological (regularity) and size (nanoscale) standards by using these materials, some difficulties were found with PMVE/MA-ES. However, the concentration of the polymers in the electrospun solution was found to be the critical point, which might be explained by changes in the viscosity of these solutions. The significant influence of the viscosity on the morphology and size during the electrospinning process has been reported elsewhere^41^.

The mixture of polymeric materials for the creation of nanostructures has the advantage of combining the positive features from each compound involved. Usually, these blends comprise a (building) polymer with the ability to form nanofibers easily and thus serving as support for the integration of other material/s with complementing activities, but which may not be suitable for use as building polymers. As previously discussed, PMVE/MA and its derivatives display a number of interesting abilities suitable for this building role, ie low toxicity, biodegradability and adhesive capacity useful as a vehicle for controlled administration of drugs in epithelia^33–38^. In this work, electrospun nanofibers were also generated by blending any of the PMVE/MA forms with fluorene-based CPEs (either HTMA-PFP or HTMA-PFNT), and therefore confirming that they can work as building polymers with assembly capacity for other compounds. The fluorescence properties of CPEs also facilitated their use as reporters to confirm the homogenous distribution, along the nanofibrous electrospun mat, of the molecular components involved and better characterize the morphology of the nanostructures.

Regarding the blends used in this study, none of these fluorene-based CPEs are capable of building electrospun nanofibers by themselves; however, using another electrospinnable polymer species, which assumes the building role, they not only allow nanofiber formation but also provide their fluorescent properties to the main structure. This means that the electrospinning process under described conditions did not modify, significantly at least, their molecular structures. Solid state fluorescence of CPE-PMVE/MA blends showed interesting emission spectra of the corresponding CPEs. In both cases, blue-shift emission occurs with respect to their corresponding spectrum in water solution. This observation has previously been linked to the possibility that the conjugate chains in the polyelectrolytes are ordered or stretched and without forming aggregates^42^. This hypothesis, together with the fact that no shift was observed for PMVE/MA-Ac nanofibers when combined with HTMA-PFP, might explain why nanofibers of PMVE/MA-Ac blended with HTMA-PFNT gave no suitable nanofibers when electrospun.

In terms of encapsulation capacity, in all synthesized nanofibers from this work high encapsulation yields of 5-ALA were obtained (97 ± 1%). In a recent study, poly(vinyl alcohol) nanofibers containing 10% w/w 5-ALA were also achieved and, although no data on encapsulation yields were given, activity values were similar between encapsulated and non-encapsulated 5-ALA^17^. The ease with which 5-ALA was encapsulated is probably due to its low molecular weight, hydrophilicity and low reactivity. Furthermore, since there were no significant differences in the encapsulation yields obtained for each type of synthesized nanofibers, the encapsulation capacity of PMVE/MA nanofibers seems not to be limited by either the PMVE/MA derivatives, or the complementary copolymers when used. However, assays to determine maximum content loads were not performed since they were focused on a fixed concentration (16.7% w/w) that approximately corresponds to the average concentration already used in approved biomedical products, Levulan® (20%) and Ameluz® (7.8%) in USA and EU, respectively.

From the pharmacological point of view, four classical kinetic release models^43–46^ were considered (zero-order (M_t_/M_∞_ = K_0_ t), first-order (Ln(M_t_/M_∞_) = −K_1_ t), Higuchi (M_t_/M_∞_ = K_h_ t^1/2^) and Korsmeyer-Peppas (M_t_/M_∞_ = K t^n^) models) in order to adjust transdermal release assay data. From these adjustments, it was found that all nanofibers diffused according to Fick’s law and loaded 5-ALA diffused in a manner more accurately described by a Higuchi model^43^, which is typified by solid flat matrices. In turn, solutions showed values of n = 0.25–0.23, ie values lower than 0.45, corresponding to a quasi Fickian diffusion, according to Dash *et al*.^46^. The stabilization before 3 h and high release rates in the first stages (30 min) observed for all release profiles make these polymers, when nanostructured as nanofibers, highly effective delivery systems for compounds such as 5-ALA, which has a half-life at physiological pH and 50 °C of about 3 h (degradation is about 25% from total 5-ALA at 32 °C)^47^. Among them, PMVE-MA-ES nanofibers appeared to be the most efficient vehicles since they reached up to 100% release of 5-ALA after 3 h.

In this sense, the quantification by HPLC of encapsulated 5-ALA, when analyzing either its release kinetics or encapsulated yields, also reveals information on the stability of the encapsulated compound. Possible modification or degradation events on the molecule may be associated to changes in the elution dynamics of the original compound. After examining and comparing the elution profiles, no differences were found between loaded and non-loaded 5-ALA, indicating that the encapsulation process and the compounds used for this purpose did not seem to affect this compound, congruent with the fact that electrospinning is, so far, the most popular methodology to create drug-loadable nanofibers^48^. These results are consistent with those obtained *in vitro* showing that encapsulated 5-ALA is able to penetrate cells and be used to produce the PpIX photosensitizer just as non-encapsulated 5-ALA is, in concordance with the results of Yoo *et al*.^17^. In addition, our study demonstrates that 5-ALA lability^47^ is prevented during long periods since its encapsulation into PVME/MA-ES nanofibers helps to maintain its stability and biological activity for at least 3 months under our storage conditions.

Consequently, we propose that encapsulation into these electrospun nanofibers might be applicable to other compounds already demonstrated to be suitable for encapsulation by PMVE/MA nanostructures^33,49,50^. Together with the fact that the biocompatibility of these polymers in nanofiber form^35–37^ was also partially confirmed in this work, since no significant effects on viability were observed when treating sensitive cell lines with them, we consider that electrospun nanofibers of PMVE/MA forms are effective vehicles for topical delivery of therapeutics. Nevertheless, further *in vivo* assays will be required in order to assess their actual potential.

## Materials and Methods

### Materials

**Figure 7:**
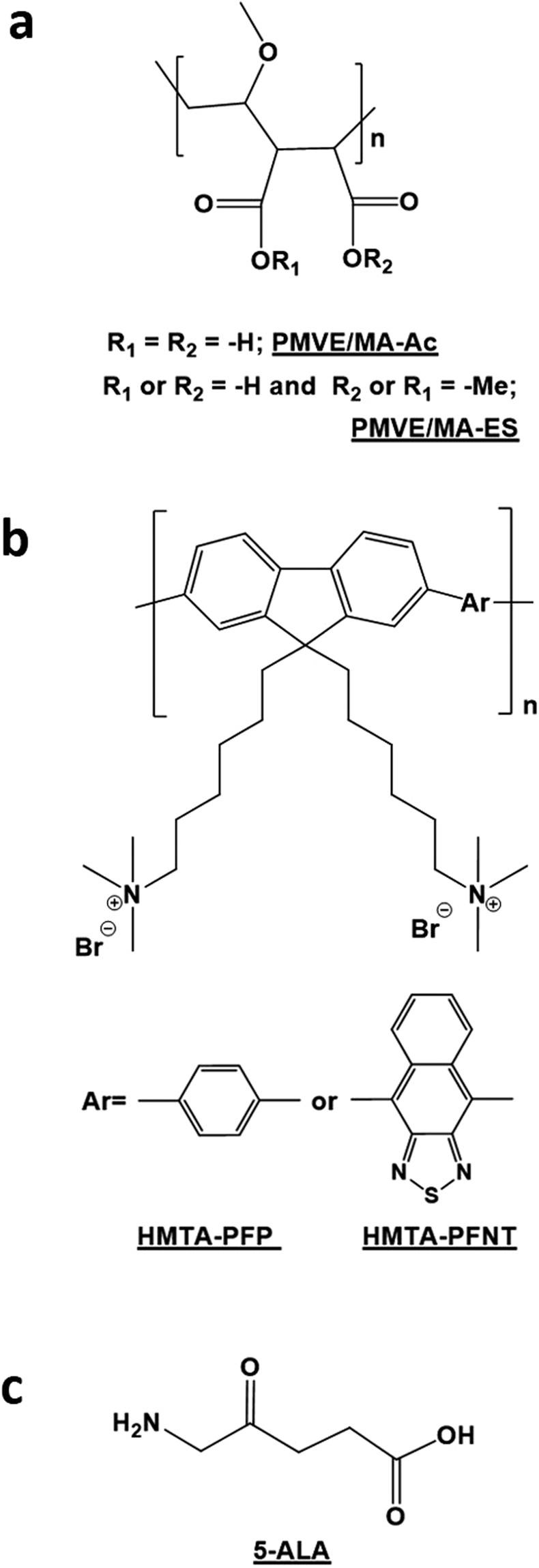
Structures of the lead compounds used. (**a**) Poly(methyl vinyl ether-*alt*-maleic acid), PMVE/MA-Ac, or poly(methyl vinyl ether-*alt*-maleic monoethyl ester), PMVE/MA-ES. (**b**) Poly{[9,9-bis(6′-*N*,*N*,*N*-trimethylammonium)hexyl]-2,7-(fluorene)-*alt*-1,4-phenylene} bromide, HTMA-PFP; poly-{[9,9-bis(6′-*N*,*N*,*N*-trimethylammonium)-hexyl]-2,7-(fluorene)-*alt-*1,4-(naphtho[2,3c]-1,2,5-thiadiazole)} bromide, HTMA-PFNT. (**c**) 5-amino-4-oxo-pentanoic acid or 5-aminolevulinic acid, 5-ALA. All these chemical structures were drawn by using ChemBioDraw Ultra v14.0.

Both fluorene-based CPEs, poly{[9,9-bis(6′-*N*,*N*,*N*-trimethylammonium)hexyl]-2,7-(fluorene)-*alt*-1,4-phenylene} bromide (HTMA-PFP, blue emitter; batch Mw: 8990 g.mol^−1^, PDI:2.0 GPC: PS calibration) and poly-{[9,9-bis(6′-*N*,*N*,*N*-trimethylammonium)-hexyl]-2,7-(fluorene)-*alt*-1,4-(naphtho[2,3c]-1,2,5-thiadiazole)} bromide (HTMA-PFNT, red emitter; batch Mw: 8340 g.mol^−1^, PDI:2.0; GPC: PS calibration) were synthesized and characterized in our laboratory as previously reported by Molina *et al*. (2009)^51^ and Kahveci *et al*. (2016)^52^, correspondingly. For the synthesis of nanofibers, both forms derived from PMVE/MA, PMVE/MA-Ac (Mw: ~216 kg.mol^−1^) and PMVE/MA-ES (Mw: ~130 kg.mol^−1^), were purchased from Sigma-Aldrich (Sant Louise, USA). The loadable pharmaceutical agent chosen, 5-ALA, was also purchased from Sigma-Aldrich. Chemical structure inputs of all these compounds, as main components of synthesized nanofibers, are shown in Fig. 7.

Cell culture material, ie 0.25% trypsin solution with EDTA, Dulbecco’s modified eagle’s medium high glucose (4.5 g/L) with L-glutamine and sodium bicarbonate (DMEM), fetal calf serum (FCS), 5000 units penicillin – 5 mg/mL streptomycin solution, phosphate-buffered saline (PBS), polystyrene culture flasks and 96-well plates, were purchased from Sigma-Aldrich as well.

MTT, ethanol, anhydrous isopropanol, hydrochloric acid and dimethyl sulfoxide (DMSO) were obtained from Merck. Milli-Q water was obtained from a Synthesis A10 system (Millipore). PpIX was purchased from Echelon Biosciences (Salt Lake City, USA).

### Preparation of solutions

Different solutions were prepared, all at room temperature (RT), depending on the materials used for each type of electrospun nanofiber synthesized. To obtain unloaded single-polymer nanofibers, 20% w/w PMVE/MA-Ac (commercialized in powder) and 25% w/w PMVE/MA-ES (commercialized as a 50% w/w solution in ethanol) solutions were prepared by dissolving in H_2_O with constant stirring during 1 h. For CPE-PMVE/MA blended nanofibers, 7.5 mg of either HTMA-PFP or HTMA-PFNT in DMSO were dissolved per gram of previously described PMVE/MA solutions. Those with HTMA-PFP were stirred for 20 h and HTMA-PFNT ones for 48 h in dark.

For the synthesis of 5-ALA-loaded (single and blended) nanofibers, all solutions were prepared as previously described for each type of nanofibers. 5-ALA (dissolved in H_2_O) was added to polymer solutions (of PMVE/MA derivates with or without CPEs) to reach a concentration of either 33.2 mg/mL or 41.5 mg/mL for PMVE/MA-Ac or PMVE/MA-ES formulations, respectively. Thus, 5-ALA final concentration was 16.7% w/w respect to the amount of both PMVE/MA derivate. All of these solutions were prepared in darkness prior to use.

To make culture medium to harvest cells, DMEM was supplemented with 10% FCS and 100 units penicillin and 0.1 mg streptomycin per mL (Sigma-Aldrich). For the assays to measure cell viability, a 5 mg/mL MTT stock solution in PBS was prepared and stored at −20 °C until used.

The lysis buffer, used for lysing and solubilizing cellular components prior to PpIX determination assays, was composed of 10 mM Tris-HCl pH 7.4, 100 mM NaCl, 1% Triton X-100, 1 mM ethylenediaminetetraacetic acid (EDTA), 10% glycerol and 0.1% sodium dodecyl sulfate (SDS).

PpIX stock solution was prepared at a concentration of 750 µM in 35% v/v HCl, from which there were prepared 7.5 µM working solutions in hydrochloric acid as well.

### Electrospinning

The electrospinning setup consisted of a disposable 2 mL plastic syringe (Becton Dickinson), which was filled with the polymeric solutions, coupled to a blunt end stainless steel hypodermic needle 316 (20 Gauge) (Sigma-Aldrich) which is connected to the positive output of a high voltage power supply Series FC (Glassman High Voltage, Inc). The flow rate was controlled by means of a syringe infusion pump KDS 100 Series (KD Scientific). A surface covered with aluminum foil, and located 10 cm from the tip of the needle, usually worked as collector system. Common glass slides (Normax, Portugal) for fluorescence microscopy and solid state fluorescence spectroscopy assays or copper TEM grids with 3 mm of diameter (Electron Microscopy Sciences, UK) for TEM analysis were coated instead for these particular applications. The polymeric solutions were electrospun at 15 kV using a flow rate of 0.5 mL/h. The thickness of the final mats was controlled by modulating the deposition time. The whole process was carried out in darkness and at RT. The relative humidity seemed not to be critical under these conditions since the process worked satisfactorily at levels ranging 35–60%. Finally, in order to evaporate solvent excess the membranes were kept in a fume hood overnight. Synthesized nanofibers were then stored protected from light at RT and dry atmosphere until used.

### Microscopy

The morphology and size of nanofibers were analyzed by TEM with a Jeol 1011 (Jeol, Japan) apparatus. The average diameter of the different types of nanofibers was obtained by using the image processing and analysis software Image J (National Institutes of Health) on TEM images. SEM images were taken with a JEO 6100 (Jeol, Japan) apparatus.

Likewise, in order to confirm and characterize the integration of CPEs to the nanofiber structure, nanofibers were observed and photographed by using a Leica DMI 3000B inverted microscope with a 63× objective and equipped with a Leica EL6000 compact light source and a Leica digital camera DFC3000G.

### Solid state fluorescence spectroscopy

The presence of CPEs in synthesized nanofibers as well as their fluorescence properties associated to imaging abilities were analyzed by solid state fluorescence spectroscopy with a QuantaMaster steady-state spectrofluorimeter (Photon Technology International). For this purpose, its solid state sample holder was set at 340° and 3.25 mm and the emission fluorescence spectra were obtained by exciting the nanofibers on a glass slide at 370 and 510 nm wavelengths. Each spectra was registered from the average of three different positions of the material.

### *In vitro* skin permeability studies

The cutaneous absorption dynamics of 5-ALA loaded into different experimental electrospun PMVE/MA types of nanofibers were analyzed *in vitro* using Franz diffusion cells (PermeGear®). Cellulose acetate membranes Cuprophan® (Medicell) were mounted on the Franz cells receptor chambers, which were connected by a 9 mm diameter (diffusional area: 0.64 cm^2^) cavity to their corresponding donor compartments. The cell receptor was filled with a 5 mL volume of 1 mM phosphate buffer (pH 7) that was in contact with the upper membrane, which was allowed to equilibrate for 30 min. Then, nanofiber mats were cut into pieces of 0.64 cm^2^, weighted, arranged facing upward into the donor and wetted with 150 µL of 1 mM phosphate buffer (pH 7). The corresponding viscous polymeric solution with 5-ALA used to synthesize each type of nanofiber was used as reference. Continuous magnetic stirring of the volume in the receptor chamber was kept at 600 rpm during the whole process. The system, comprising six Franz cells, was connected to a thermostatic bath (Selecta Digiterm-100) which maintained temperature at 32.0 ± 0.5 °C like the temperature at the skin surface^53^. An aliquot of 400 μL in the receptor compartment was removed at each settled time-point after drug application. Withdrawn volumes were immediately replaced with fresh receptor buffer. This assay was performed twice for all nanofiber types using a different production set each time. Samples were analyzed by HPLC immediately after their collection to avoid 5-ALA degradation in polar solutions.

### HPLC analysis

In order to assess the amount of 5-ALA loaded into the nanofibers and diffused in the skin permeability assays as well as any degradation sign of the molecule, the detection of 5-ALA was optimized using HPLC with a Merck/Hitachi HPLC system and an Alltech 3300 Evaporative Light Dispersion (ELSD) detector (Grace Davison Discovery Sciences) coupled to it (ELSD-HPLC). The isocratic mobile phase used was 98% H_2_O with 0.1% v/v trifluoroacetic acid and 2% acetonitrile with 0.1% v/v trifluoroacetic acid, a flow rate of 1 mL/min and an injection volume of 10 μL, similarly as it is described in previous studies^54^.

By using these settings, a standard curve of detected 5-ALA, previously dissolved in 1 mM phosphate buffer (pH 7) with final concentrations ranging 20–250 μg/mL, was performed by ELSD-HPLC before running the experimental samples. A quadratic correlation was found between concentration and detected peak area in the range of selected concentrations with regression coefficient values (R^2^) of greater than 0.999 (Supplementary data, Fig. S3A). Samples obtained from the skin permeation assays were used without any further preparations. To assess the amount of 5-ALA loaded into the each type of electrospun nanofiber by ELSD-HPLC, 1 cm^2^ pieces of nanofiber mats were cut, weighted and dissolved with up to 200 µL of 1 mM phosphate buffer (pH 7) previously (n = 3). The drug concentration was measured as µg of detected 5-ALA per µg of total theoretically loaded 5-ALA and represented as percentage.

### Cell culture

The well-known immortalized human keratinocyte cell line HaCaT (gently provided by Prof. Antonio Ferrer) and the human adenocarcinoma cell line SW480 (ATCC® Ref.: CCL-228™) (gently provided by Dr. Miguel Saceda) from colon tissues, were used for the *in vitro* biological assays of this work. The culture media, previously described in section 2.2, was used for all cell lines. Likewise, all of them were grown in 25 cm^2^ flasks at 37 °C in a humidified 5% CO_2_ air atmosphere.

### Cytotoxicity assay

The potential toxicity on tested cell lines of all combinations of nanofibers as well as of their components individually was analyzed by measuring the changes in cell viability by MTT assay. Briefly, cells at 90–100% confluence, which were seeded in 96-well plates 24 h before, were treated with different concentrations of each type of nanofiber and compound (previously dissolved in 1 mM phosphate buffer (pH 7)) in cell culture media for 24 h (100 µL/well). Then, treatments were replaced with fresh cell culture media (100 µL) with 0.5 mg/mL of MTT (from 10 × concentrated stocks). Cells were then incubated for 2 additional hours, media carefully removed and the colored formazan product was dissolved in 100 µL of DMSO and measured at 570 nm (reference at 620 nm) with a SPECTROstar® Omega absorbance microplate reader (BMG LABTECH, Germany). The optical density was directly correlated with quantity of cells and expressed in percentages relative to control group (untreated cells). All experiments were performed in triplicate and results are shown as means with standard deviation (sd) from three different experiments.

### PpIX accumulation assay

5-ALA is the precursor of the photosensitizing agent PpIX, the ultimate responsible for the production of the cytotoxic reactive oxygen species (ROS) and singlet oxygen in PDT^55^. In order to check the functionality of the 5-ALA loaded into the different types of nanofibers, the amount of PpIX converted from that 5-ALA internalized into cells was measured as previously described by Jin Ju Yoo *et al*.^17^. To this end, cells at 90–100% confluence, which were seeded in 24-well plates 24 h before, were treated with different concentrations of each type of nanofiber and 5-ALA (previously dissolved in 1 mM phosphate buffer (pH 7)) in cell culture media for 24 h. Then, cells were lysed with 100 μL/well of lysis buffer, dispensed in black 96-well plates and the accumulated PpIX in cells was measured with a Cytation 3 cell imaging multi-mode plate reader (BioTek Instruments Inc., USA) at 412 nm (excitation) and 665 nm (emission) wavelengths (optimal values for PpIX fluorescence detection (see Fig. S2 in Supplementary data for further details). A standard curve correlating cell-accumulated PpXI emission units with initially added 5-ALA (concentrations ranging 0–200 μg/mL) was performed and a linear correlation was found within the range from 0 to 100 μg/mL, with regression coefficient values (R^2^) higher than 0.95 for both cell lines in all experiments (Fig. S3B in Supplementary data). The sensitivity of the methodology was assessed by adding a gradient of concentrations in lysis buffer of PpIX (Supplementary Fig. S3C). All experiments were performed in triplicate and results are expressed in arbitrary units (a.u.) and shown as means with sd from three different experiments.

### Statistical analysis and graphics

Data were statistically analyzed by either unpaired *t*-test or one-way ANOVA (depending of the variables of the experiment). GraphPad Prism v5 software was used for performing the statistical analysis. Both GraphPad Prism v5 and Microsoft Excel were used for creating the graphs.

### Data availability

All data generated or analyzed during this study are included in this published article (and its Supplementary Information files).

## Electronic supplementary material

Supplementary Information
