# Supplementary material for: Poly(methyl vinyl ether-alt-maleic acid) and ethyl monoester as building polymers for drug-loadable electrospun nanofibers
Source: Sci Rep. 2017 Dec 8;7:17205. doi: 10.1038/s41598-017-17542-4 (PMC5722912; doi:10.1038/s41598-017-17542-4)
Supplement: Supplementary file 1 — Supplementary Information [file 41598_2017_17542_MOESM1_ESM.pdf]

**Poly(methyl vinyl ether-*alt*-maleic acid) and ethyl monoester as building polymers for drug-loadable electrospun nanofibers**

Amalia Mira, C. Reyes Mateo, Ricardo Mallavia, Alberto Falco

# SUPPLEMENTARY INFO

**Figure S1**

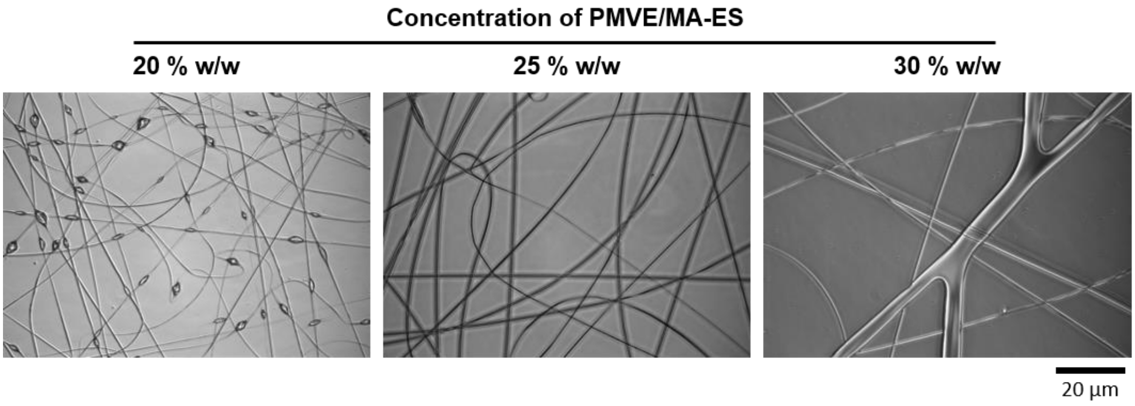


**Figure S1. Optical microscopy images of nanofibers obtained from electrospinning solutions with different concentrations of PMVE/MA-ES.**

**Figure S2**

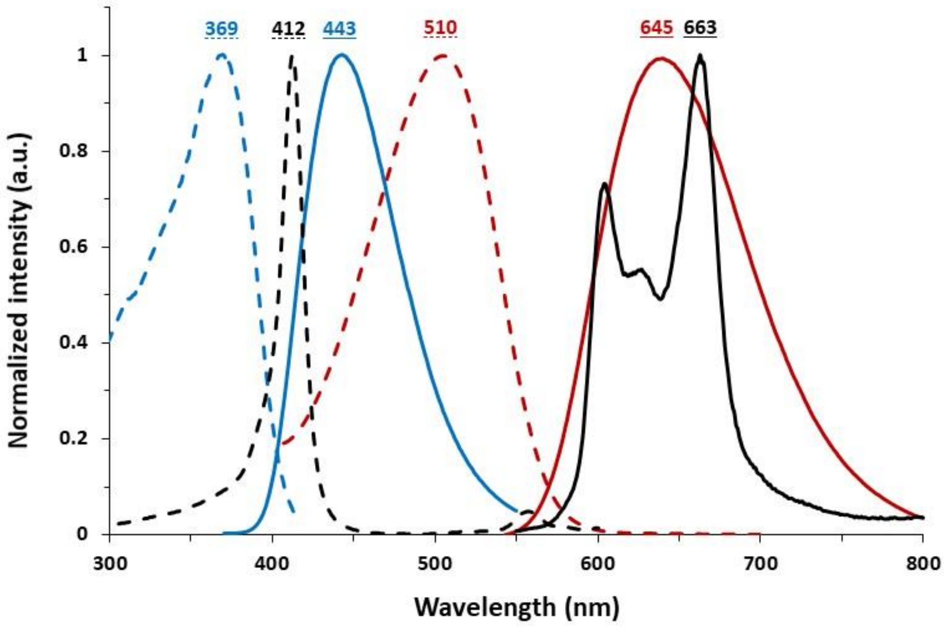


**Figure S2. Spectroscopy analysis of PpIX and CPEs.** Normalized fluorescence spectra of PpIX (black lines) in HCl solution and HTMA-PFP (blue lines) and HTMA-PFNT (red lines) in water solutions. Absorbance spectra are drawn in dash lines and emission spectra in solid lines. Highest values are indicated on the top of each peak.

**Figure S3**

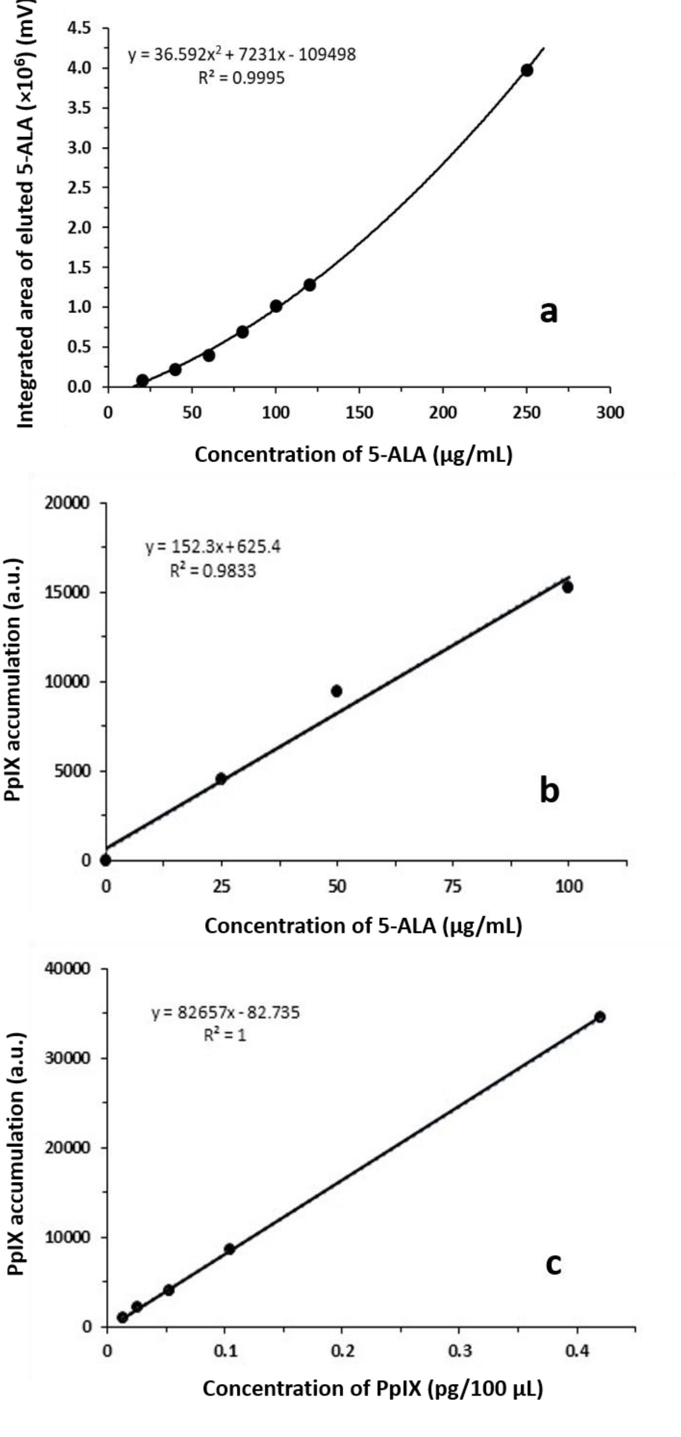


**Figure S3. Representative calibration curves used in this study.** a) Calibration curve of detected 5-ALA in HLPC elutions and used for release-kinetics studies. b) Calibration curve of intracellular PpIX levels detected, with Cytation3 plate-reader, from 5-ALA treated cells. c) Calibration curve of PpIX levels detected, with Cytation3 plate-reader, from PpIX solutions.
